# Role of Immersive Virtual Reality in Motor Behaviour Decision-Making in Chronic Pain Patients

**DOI:** 10.3390/brainsci13040617

**Published:** 2023-04-05

**Authors:** Javier Guerra-Armas, Mar Flores-Cortes, Consolacion Pineda-Galan, Alejandro Luque-Suarez, Roy La Touche

**Affiliations:** 1 Faculty of Health Sciences, Universidad Las Palmas de Gran Canaria (ULPGC), 35016 Las Palmas, Spain; 2 Faculty of Health Sciences, Universidad de Malaga, 29071 Malaga, Spain; 3 Instituto de la Investigacion Biomedica de Malaga (IBIMA), 29071 Malaga, Spain; 4 Departamento de Fisioterapia, Centro Superior de Estudios Universitarios La Salle, Universidad Autónoma de Madrid, 28023 Madrid, Spain; 5 Motion in Brains Research Group, Institute of Neuroscience and Sciences of the Movement (INCIMOV), Centro Superior de Estudios Universitarios La Salle, Universidad Autónoma de Madrid, 28023 Madrid, Spain; 6 Instituto de Dolor Craneofacial y Neuromusculoesquelético (INDCRAN), 28008 Madrid, Spain

**Keywords:** virtual reality, body embodiment, motor behavior, chronic pain, decision-making

## Abstract

Primary chronic pain is a major contributor to disability worldwide, with an estimated prevalence of 20–33% of the world’s population. The high socio-economic impact of musculoskeletal pain justifies seeking an appropriate therapeutic strategy. Immersive virtual reality (VR) has been proposed as a first-line intervention for chronic musculoskeletal pain. However, the growing literature has not been accompanied by substantial progress in understanding how VR exerts its impact on the pain experience and what neurophysiological mechanisms might be involved in the clinical effectiveness of virtual reality interventions in chronic pain patients. The aim of this review is: (i) to establish the state of the art on the effects of VR on patients with chronic pain; (ii) to identify neuroplastic changes associated with chronic pain that may be targeted by VR intervention; and (iii) to propose a hypothesis on how immersive virtual reality could modify motor behavioral decision-making through an interactive experience in patients with chronic pain.

## 1. Introduction

Chronic pain has been classically defined as pain that lasts or recurs for longer than three months [1]. This definition is based on a purely temporal criterion. However, since the last IASP classification (ICD-11), chronic pain is now considered a condition itself [2]. Chronic primary musculoskeletal pain is defined as CPP located in musculoskeletal areas that may present with spontaneous or evoked pain in the affected region, accompanied by allodynia and/or hyperalgesia [3].

The understanding of how people experience pain as well as the strategies to address this condition is challenging. Some authors have reflected on the need to embrace the complexity of the chronic pain experience [4], in which the relational dynamics among pain determinants are a crucial cornerstone for its understanding [5]. Pain can be considered part of an overall protection system, a body-world defence mechanism. In the presence of pain, a deviation from potential future actions that may be perceived as self-threatening may be necessary [6,7].

A first-person perspective is imperative to understand the experience through and within a body in a particular space and time [8]. Chronic pain can dramatically impact the interplay between the subject and the world, as it permeates all aspects of a person’s life. When the pain and suffering become chronic, they become embodied, i.e., part of the self [8]. Therefore, people in pain often feel alienated from their bodies and their surroundings simultaneously [9]. 

Several models have attempted to explain the pain processing and motor behavior experienced by a person with chronic pain and their relationships with the environment through sensory information, both from within and outside the body (Table 1). However, none of these models alone have been able to answer all the questions that have arisen in our understanding of the experience of chronic pain and its underlying mechanisms. If it were possible to achieve this, much more needs to be done to get a unified theory of pain [10]. 

Among possible options for chronic pain patients; VR systems can be tailored to individual needs to provide therapeutic interventions within a functional and motivating context in a manner that alters the subjects’ experiences [16]. As an example of a VR intervention for patients with chronic shoulder pain, a graded shoulder flexion task can be proposed using gamified software in a virtual simulation of a real environment (for example, a kitchen where objects are placed on a shelving unit) or a science fiction simulation (for example, in space, shooting at alien spaceships) to reduce fear-avoidance behavior in this movement. This may be key to the appropriate selection of the best individual therapeutic approach within patient-centered care, which has been identified as fundamental in the management of people with chronic pain conditions [17].

### An Introductory Review to VR Effects on Chronic Pain

Virtual reality (VR) refers to simulated experiences with multisensory content (visual, auditory, haptic, etc.), intentionally presented to the individual’s senses [18]. In recent years, research and clinical application of virtual reality in pain management have substantially increased [18], in both acute and chronic pain [19]. Although there are different modalities of virtual reality, features such as presence and immersion associated with immersive virtual reality [20] can represent an ideal medium for non-pharmacological management of chronic pain [21,22]. The integration of evidence-based information may serve to build a new and more comprehensive conceptual framework on the neurophysiological mechanisms of immersive virtual reality than previous ones [23].

Initially, we intend to establish the state of the art on the effects of VR on chronic pain. To address this, a literature search was conducted, collecting the results of different systematic reviews and meta-analyses published in the last five years. Regarding the search strategy, we searched for systematic reviews using PubMed (from 2017 to 26 November 2022). With respect to the eligibility criteria, the selection criteria used in this review were based on PICOT-criteria for “population: chronic pain patients”, “intervention: virtual reality or exergame”, and “study design: systematic review and/or meta-analysis” (Table 2).

The search strategy used on the PubMed database was: ((“Virtual Reality/Exergaming “[Mesh] AND “Chronic pain” [Mesh]) OR ((“Virtual reality” AND “Chronic pain”)) AND ((systematic[sb] OR Meta-Analysis[ptyp]). We included all eligible articles written in English with full-text available.

The data extracted from each study were as follows: title, search methodology, number of included studies, patient population, and intervention(s); if applicable, outcome(s) and/or outcome measure(s); if applicable, follow-up period(s), results, limitations, and conclusion(s). Regarding the results, Appendix A summarizes all the findings, with eight reviews included.

The evidence from systematic reviews and meta-analyses suggests that VR reduces pain intensity and improves function in patients with chronic pain. However, the heterogeneity in the type of VR and software applied, the dosimetry of the treatment, and the outcome measures collected show the need to carry out studies with higher methodological quality.

Although the evidence supports the use of VR, some studies reported that prolonged and continuous exposure to VR could cause a disorder called “Cibersickness”, characterized by dizziness, headache, nausea, postural pain, or disorientation [24]. The rate of side effects in VR is still very variable, although generally low, due to the influence of factors related to both the type of device used and the characteristics of the software [25,26]. Further, the etiopathogenic mechanisms of these side effects are not yet known; as such, it is hypothesized that virtual reality might cause a conflict in multisensory integration [26]. No other significant adverse effects have been reported, showing that VR is a safe intervention.

The hypoalgesic mechanisms underlying VR are multifactorial, being mediated by the different dimensions of the pain experience: sensory-discriminative, affective-motivational, evaluative-cognitive, and motor behavior [27,28]. Nevertheless, the growth in scientific research has not been accompanied by substantial advances in the understanding of how VR influences the experience of pain [18] and which mechanisms may be involved in the clinical effectiveness of these interventions in people with chronic pain. The combination of current evidence from musculoskeletal research, pain neuroscience, and behavioral sciences may help to (i) understand and (ii) guide the application of VR to chronic pain patients [29]. The present narrative review proposes a new hypothesis based on how VR could modify the dynamic relationship between multisensory integration, body embodiment, sensorimotor performance, and motor reinforcement learning through an interactive experience in people with chronic pain.

## 2. Immersive Virtual Reality as a Medium for Altering Sensorimotor Decision-Making in Chronic Pain Patients: A Neurophysiological Hypothesis

**Figure 1:**
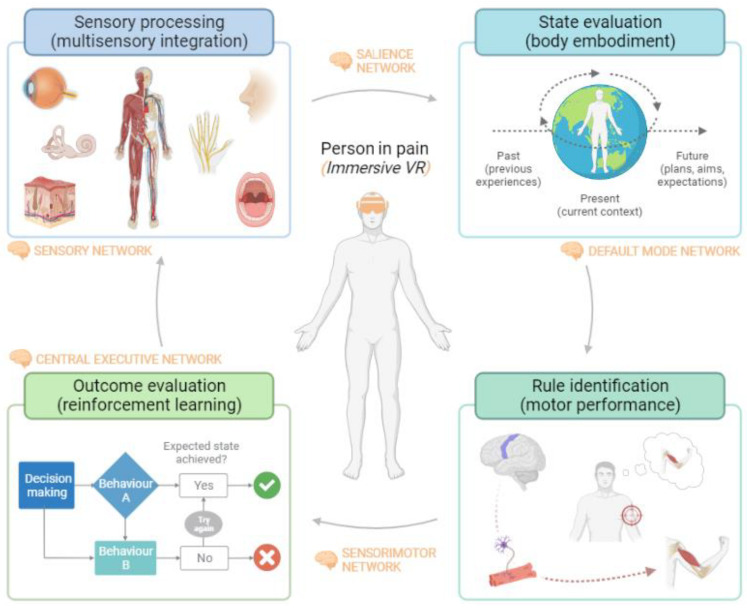
Schematic representation of sensorimotor decision-making and how virtual reality can impact brain networks related to it. A four-stage model of the dynamic relationship between motor behavior decision-making in people with chronic pain and immersive virtual reality intervention is presented. In the first phase, sensory information (visual, auditory, and haptic) provided by VR may impact both the sensory network and the salience network, modifying the relevance of the stimulus and the integration of sensory modalities. Subsequently, this new information could influence the person’s body image and their relationship to the environment associated with the default network. In this process, VR can modulate different features of body image (ownership and agency) through the body illusion induced by full-body virtual avatars. The congruence between the movements of this avatar and those of the patient can generate a new motor learning process associated with the sensorimotor network, which could modulate pain-associated motor responses and behaviors such as movement-evoked pain. Finally, the change in valence of the perceived threat of a motor task and its influence on pain-related executive processes may trigger reinforced motor re-learning processes. The safe context provided by VR could facilitate the optimization of motor behavior, thus enhancing the relationship between the individual and the environment and his or her own body, by influencing these four stages described above, which were created with Biorender.

A framework with four different processing levels that pertain to sensorimotor decision-making to estimate the risks and manage the threats appropriately has been proposed [26,27]. Our proposal is that VR embeds the person in pain into a different environment that may modulate the individual’s subjective experience of pain and his or her relationship with the environment by modifying the dynamic interplay between brain networks related to motor behavior in chronic pain patients (Figure 1) [11]. Such influence can be found in all four phases of decision-making:

1. New visual and auditory information provided by immersive virtual reality is integrated with other sensory stimuli and reaches the sensory cortex, which is associated with the first threat evaluation (sensory network) [30]. Activation of the sensory pathways triggers activation of the amygdala and insula, which are associated with the valence of the stimulus and the distress experienced by the person (salience network) [31].
2. A change in the afferent sensory information can impact the current state evaluation of the environment and body image (the default mode network) [32]. This could modulate the bidirectional and inversely related link between the saliency network and the default mode network, allowing body image to be influenced by full-body virtual avatars.
3. If the virtual environment is safe enough and does not present cues of threat, goal-directed motor commands will be activated instead of protective/defensive behaviors (sensorimotor network) [33]. That is particularly necessary in people with chronic pain, where altered motor behavior is common.
4. Moving a person in pain in a safe environment can stimulate downward modulation of pain and promote analgesia [34]. Breaking the negative expectation of pain during movement can facilitate the extinction of fear memories acquired through contextual fear conditioning (the central executive network) [35,36]. The maintenance of this non-threatening environment could replace the fear memory with a new safety memory related to movement. This framework suggests that VR could be a potential tool to produce motor relearning in the context of pain [37].

Nevertheless, this process is affected by a great number of uncertainties [38], from sensorimotor noise to ambiguity about the environment [39,40]. For an overview of the influence of uncertainty in sensorimotor decision making, see Bach et al. [40].

Next, we discuss the different stages of decision-making for motor behavior within the experience of chronic pain. How these components create a recursive loop that feeds back and shapes the individual’s dynamic interplay with their internal and external environment [41]. In relation to each component, we reflect on the impairments found in chronic pain patients and the possible effects of VR on them.

### 2.1. Multisensory Integration Alterations in People with Chronic Pain

Gifford’s Model of mature organism (MOM) proposed how our nervous system allows us to use sensory information to gain knowledge about the state of our body and the world around us, which affects the way we behave in a perceived threat situation [10]. The concept of multisensory integration explains the convergent processes of sensory modalities (e.g., auditory, visual, tactile, proprioceptive, and nociceptive modalities) required to generate a unified and coherent perception [42]. Perceptual experiences are made possible by a dynamic integration of sensory signals (multisensory processing) from different modalities that are constantly updated to encode the representation and configuration of the body and its relationship to the environment [43]. Similarly, sensory information does not only include exteroception but also interoception [43]. 

The influence of multisensory integration on nociceptive processing has been previously described [44]. Many pathological pain conditions are accompanied by reduced accuracy of sensory inputs and problems with filtering irrelevant information [45]. More specifically, localized chronic musculoskeletal pain is associated with sensory disturbances at or near the painful region [46,47], and there is evidence that sensory deficits may precede pain [48]. Similarly, chronic pain has been commonly associated with reduced tactile sensitivity [45,46] and with disorganization in the somatosensory cortex [11]. Additionally, impaired tactile acuity and a greater somatosensory temporal discrimination threshold compromise the quality of the sensory information available to the nervous system, which would contribute to sensorimotor conflict [49]. Recently, low somatosensory cortex activity has been linked as a possible neurophysiological mechanism in the transition from acute to chronic pain in patients with low back pain [50]. Preliminary evidence in people with chronic pain has found discordances between motor output and peripheral feedback [51] and between efferent pathways from the motor cortex with the afferent feedback to the primary sensory cortex [52]. 

In patients with chronic pain, there are shifts in the excitability and connectivity of neural networks from areas more related to sensory processing to areas linked to affective–emotional processing. In addition, salience networks have been observed [31]. The corticolimbic system seems to play an important role in the development, maintenance, and amplification of chronic pain and is associated with affective aspects of pain and regulates emotional and motivational responses [53]. Increased information transfer between somatosensory cortex and salience network regions, particularly the anterior insula, likely plays an important role in re-allocating attentional focus and affective coding of somatic nociceptive afference from specific body areas [30]. Within the salience network, the prefrontal cortex (PFC) and nucleus accumbens (NAc) have been identified as part of an “gate-keeping” mechanism that estimates the relevance of a stimulus (valence) and modulates information flow in the descending inhibitory pain system [54]. Valence refers to the categorization of a stimulus as pleasant (positive valence) or unpleasant (negative valence) in the context of affect and emotion [44]. The impact of cognitive-emotional factors on the onset and/or persistence of chronic musculoskeletal pain has been previously studied [55,56].

Moreover, there is evidence that thalamo-cortico-basal ganglia loops integrate sensorimotor, affective, and cognitive information, which may be related to the pain experience [54]. According to the triple network model, this may indicate the dynamic and complex interrelationships between different central networks (somatosensory, salience, default mode network, sensorimotor, and central executive) in the person with chronic pain [11].

#### How VR Can Improve Multisensory Processing

Through stimulating the visual, auditory, and sensorimotor networks, VR may modulate pain perception [57]. The hypoalgesic effects of VR have been attributed to competition for the limited attentional resources shared between sensory afferents provided by VR and incoming nociceptive signals [58]. Interestingly, the visual information that a person has about their body increases the spatial acuity of touch [59,60] and reduces the perceived intensity of acute pain [61,62]. VR application in pain patients increases activity in areas associated with pain inhibition [63]. Some authors hypothesize that VR acts through the periaqueductal gray (PAG), anterior cingulate cortex (ACC), and orbitofrontal cortex to divert attention from pain, modulate the activity of the descending pain control system, and influence pain perception [64].

As an example of a virtual reality-induced change in multisensory integration, the rubber hand illusion (RHI) describes a phenomenon in which participants experience a rubber hand as being part of their body using the synchronous application of visuo-tactile stimulation to the real and the artificial limb [65,66]. Visuo-motor (VM) and visuo-tactile (VT) synchronous stimulation by RHI is important to induce virtual body illusions (BOIs) [67]. Hypoalgesic effects through virtual body manipulation have been previously studied, showing the relationship between multisensory integration, body image, and pain perception [68].

By modifying sensory processing in immersive VR, we may modulate the relevance of the stimulus; inducing a positive valence, which will interfere with the functioning of the salience network as well as the interlinked networks that may be involved in the experience of pain [11]. The interplay between sensory and cognitive–affective mechanisms opens the possibility of modifications that target body perception disturbances in people with chronic pain [52,69]. 

### 2.2. Body Perception Disturbance in Chronic Pain Patients

The importance of the somatosensory system for tactile recognition, body perception, and motor actions has been previously described [70]. Our body image (BI) is continuously updated based on the stimuli that the person receives [71], and this updating can be seen as an adaptive strategy of response to the outside world [72]. This sensory process, called somatoperception and somatorepresentation, involves the activity of both sensory and self-representation brain networks [70,73]. These somatosensory networks can lead to the emergence of different properties related to body perception (Table 3) [70].

Body embodiment refers to the self being situated in the environment and emerging inseparably from bodily information [74]. This phenomenon emerges from a complex interaction between bottom–up and top–down signals [71] and depends on the integration of multiple sensory modalities, including vision, hearing, touch, and proprioception [75]. The sense of embodiment must comply with the three sub-components (co-location, ownership and agency).

The body’s embodiment may entail activation of the brain’s default mode network (DMN) [76], which controls self-representational processing. According to the triple network model of pain, this network (DMN) is important in chronic pain [77]. The longer the pain persists, the stronger are the links between the primary somatosensory cortex and the default mode network. This connection has been found to be more active in people with complex regional pain syndrome, chronic low back pain, and osteoarthritis [32]. Yet, this pathway may occur in both directions, with a change in one area influencing the other [78]. 

Chronic pain is accompanied by a variety of body perception disturbances [79]. Indeed, people in pain often exhibit distortions in their perception of the positions and sizes of the affected body parts [52,80]. Moreover, altered processing in the premotor and somatosensory networks has been associated with the feeling of disownership over one’s own limbs in participants with body perception disturbance [81]. Therefore, contradictory sensory signals from one part of the body can alter the cortical somatorepresentation of that body [82]. These distorted representations of the body have also been linked to changes in the activity of the primary motor cortex and somatosensory cortex, which thereby result in sensorimotor incongruence [40].

#### How VR May Impact Body Embodiment

Attentional distraction has been the main proposed mechanism of VR for the reduction of acute pain [83], but these short-term positive effects of distraction appear less relevant when pain persists [84]. In contrast, immersive VR allows the “replacement” of a person’s real body, enabling the subject to feel embodied in a virtual body from a first-person perspective [71]. Hence, redefining the subjective experience of the virtual embodiment may produce alterations in bodily sensations [85]. It has been proposed that multisensory interventions may be effective at improving distorted body images [86] and may induce positive plastic reorganization of the somatosensory cortex [87] in cases of somatorepresentation distortion [69,73].

Body Illusions (BOIs) refer to altered perceptual states where the perception of the body image significantly differs from the actual physical body, for example in aspects such as size, shape, posture, location, or sense of ownership or agency [88]. Thus, BOIs arise as a result of the brain’s predictive processing of all incoming sensory signals about one’s own body [83,89]. In this sense, different studies have shown that there is a bi-directional relationship between body perception and pain perception [90]. Experimental research has investigated if the manipulation of the characteristics of the avatar’s virtual body may influence the physiological, cognitive, and behavioral responses to threat and pain sensitivity of the subjects [68,71,91]. Although the visualization of changes in the morphological features of the virtual body can influence the perception of pain, these changes should be adapted to the specific characteristics of patients with chronic pain [92]. 

Immersive VR might be useful in rehabilitation not only to modulate the perception of pain but also for motor-related functions and body perception disturbances [91] in healthy and clinical populations [48,71]. For instance, Harvie et al. investigated the short-term effects of embodying superhero-like full-body avatars on pain and body image in people with chronic low back pain [84,93], which had a favorable effect on body image. 

Virtual full-body illusions can be easily accomplished by coupling the participant’s movements with the avatar without the need for additional tactile stimulation via visuomotor congruence [74,94]. The sense of agency has been described as a crucial aspect for the coherence of the internal representation [95] and has been linked to premotor and parietal areas involved in generating motor intentions and subsequent action monitoring [76]. This could have potential for VR motor re-learning strategies, as has also been suggested in neuro-rehabilitation [96].

### 2.3. Pain-Related Movement Dysfunctions

A broad range of changes in motor-related functions (sensorimotor processing, motor deficits, and body perception disturbances) can be found in people with chronic musculoskeletal primary pain [48]. In fact, up to 75% of people with chronic pain report that pain negatively impacts their ability to exercise, lift objects, or walk [97]. According to the sensorimotor theory of pain, there might be an incongruence between motor intention and specifically tactile and proprioceptive sensory feedback in painful conditions even in the absence of tissue pathology [98,99]. The activation or “firing” of silent nociceptors in response to movement-related stimuli that are not normally painful has been described as a multisensory phenomenon called movement-evoked pain (MEP) [100]. For example, sensitivity to movement-evoked pain and pronociceptive profiling have been observed in people with chronic shoulder pain [101]. Both nociception and pain may influence goal-directed sensorimotor performance at multiple levels [102], from setting task goals, action selection and planning, movement execution, and feedback mechanisms. Similarly, the impact of sensorimotor processes on central brain regions involved in nociceptive processing and motor analgesia has been described previously [33,103].

Pain-related movement dysfunctions describe long-term motor behaviors in the presence or anticipation of pain [102]. According to the embodied predictive processing theory of pain, this phenomenon involves central and peripheral neurophysiological mechanisms that determine pain-associated behavior [12,28]. Where the anticipation of pain becomes a learned behavior [104], in which any motor behavior can be triggered as a predictive cue for pain [105,106]. However, pain-related movement dysfunctions should not be seen as a deflection of the ideal movement but rather promote adaptive behavior embedded in a threatening context [107]. Nevertheless, these adaptive motor strategies may often have negative long-term consequences, and it is therefore necessary to seek motor relearning strategies to avoid these consequences [108].

#### How VR May Enhance Sensorimotor Performance

Optimal sensorimotor performance includes the ability to gradually modify our motor commands to compensate for changes in our body and the environment [109]. Neuroimaging studies have also shown that visual and somatosensory processing are involved in guiding actions [70]. In this regard, the primary motor cortex is interconnected with descending inhibitory pain regions and sensory processing areas [103]. VR could play a role in motor function by modifying visual and somatosensory information, as previously explained.

Virtual reality could be classified as a form of visual feedback therapy, similar to mirror therapy [110], and may share similar mechanisms of action with movement representation strategies such as motor imagery (MI), action observation training, and visual feedback therapy [111]. Neurophysiological theories supporting the effects of visual feedback therapy are based on the possible improvements during the integration of sensorimotor processing and the adaptive cortical reorganization through the mirror neuron system [111]. Movement representation strategies have been proven to be effective in relieving chronic musculoskeletal pain [112,113]. For instance, VR mirror therapy has been found to affect nociception and reduce the activation in cortical limbic and default mode regions, attenuating the affective sense-of-self, internal body perception, body-related (interoceptive) internal representations, and attention to nociceptive signals [114]. Movement representation techniques may also trigger the activation of areas related to the planning, generation, and adjustment of voluntary movement at the neurophysiological level [115]. Similarly, these techniques down-modulate hyperactivity in the somatosensory cortex, increase intracortical inhibition, and increase impaired intraparietal processing, reflecting impaired body representation [116].

According to a review conducted by Pyasik et al. [91], the movements of the embodied virtual avatar significantly affected the participants motor functioning, especially when the movements were performed synchronously and actively by the patient. It has further been proposed that the mirror neuron system may be more active when the observed movements are part of one’s motor repertoire than when the observed movements are not part of one’s motor repertoire [74]. Hence, activation patterns in motor networks evoked by real and virtual movements were largely comparable when using an immersive VR system in healthy patients [117]. 

Virtual reality exercise is likely to promote effects similar to those of traditional exercise [118]. Exercise-induced hypoalgesia (EIH), which has been defined as a generalized reduction in pain and pain sensitivity that occurs during exercise and for some time afterward, may also be mediated by the immune, pain modulation systems (opioid/serotonergic), and mesocorticolimbic systems [119,120,121]. Preliminary evidence on active virtual reality EIH shows an added hypoalgesic effect in a healthy population [122,123,124]. However, exercise may not only restore the functioning of the mesocorticolimbic system in the chronic pain state but may also facilitate the extinction of acquired pain-related fear memories [125]. Thus, the immersive context of VR might help “trick the brain” through the mirror neuron system and change the self-perception of patients, reduce pain perception in exercise, and enhance motor cortical excitability and faster movements during motor training [94].

### 2.4. Threat Learning in Chronic Pain

Pain serves as a protective mechanism, leading to changes in motor behavior. If maintained, altered movement can contribute to poor recovery, disability, and decreased quality of life [98]. Even though nociceptive pathology has often long subsided, chronic musculoskeletal pain patients have typically acquired a protective (movement-related) pain memory [126] to potentially take action to avoid harm [127]. This “protective memory of pain” might have been acquired through reinforcement learning, which refers to the ability of learning the associations between stimuli and the occurrence of pleasant events, called rewards, or unpleasant events, called punishments [104]. This process has been identified as key to the learning of pain-related behaviors [128]. 

Chronic back pain has been associated with altered threat learning, differentiating less between threat and safety cues than in pain-free individuals [129]. It seems that the sensorimotor systems of people with pain might not fully correct for and/or adapt to conflicting information [48]. For instance, a distressing pain experience seems to motivate individuals to adjust goal-directed behaviors that maximize their rewards during a task, suggesting that ongoing pain facilitates emotional decision-making behaviors [130]. Moreover, disruption of salience processing may contribute to positive (spontaneous and evoked pain) and negative symptoms (catastrophizing, fear, or altered cognitions) [131]. The link between inappropriate pain-related beliefs and altered motor behavior in chronic pain people has already been discussed [132]. However, a decrease in pain-related fear has been shown to reduce movement-related pain without modulating avoidance movements in the lumbar spine [133]. Furthermore, it has been observed that alterations in motor behavior in patients with chronic low back pain are associated with task specificity and not so much with general pain-related fear [134]. Therefore, the value of the threat has a relevant weight in the valence (positive or negative) of the task and therefore in the activity of the salience network [135]. A negative valence may have a relevant role in threat learning in chronic pain [37] and pain-related movement dysfunctions.

#### How VR Can Induce Motor Reinforcement Learning

Evidence shows that VR may also produce positive effects on the cognitive-affective dimension of pain and may reduce kinesiophobia, pain-related fear, and anxiety [136,137]. The effects of BOIs on higher-level cognitive processes (perception, motor functions, executive functions, personality, and social cognition) have been proposed [91]. Furthermore, descending influences from higher structures (top-down), such as the cortico-limbic system or hippocampus, should also be considered in pain modulation systems [138].

Movement-based therapies should aim to guide patients to increase their perception of the environment by providing different possibilities [139]. The goal of cognition-targeted exercise therapy is to decouple movement-related threat perception. Moreover, exposure therapy triggers a new memory of safety by replacing or bypassing the old movement-related pain memories [126]. In this sense, VR may create specific contexts to maximize the mismatch between expectations and actual experiences to optimize inhibitory learning [36].

An enactive-biopsychosocial framework may help in understanding how body-based and movement therapies are ways to alter subjects’ experiences [14,139]. Virtual environments may provide a non-threatening context [140] for safety learning [141] and can be used to offer a personalized motor learning experience for patients with chronic pain [16]. Affordances are a part of the enactive-biopsychosocial model and mean the interactive opportunity that a virtual environment offers to an embodied organism to modify its behavior through a new sensorimotor learning opportunity [139,142]. VR provides new affordances [57] by “training” ourselves to find solutions to motor problems in a task-specific manner [16], including meaningful and challenging daily living activities [16]. For an analogy between the affordance-based model of chronic pain and a video game, see Coninx et al.’s recent paper [143].

## 3. Conclusions

**Figure 2:**
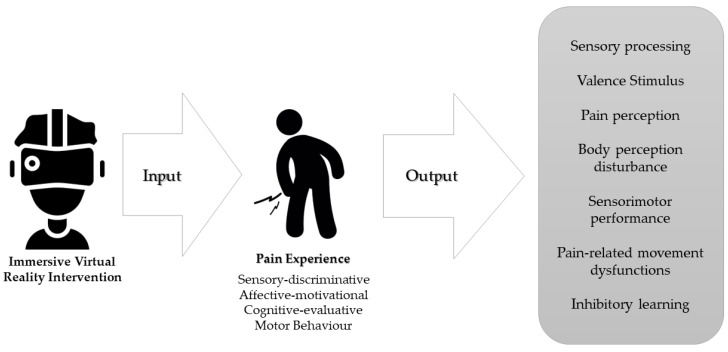
Therapeutic targets of immersive virtual reality intervention according to the distinct dimensions of the experience of chronic pain.

In conclusion, neuroplastic changes associated with chronic pain [11] may be a therapeutic target for VR-based interventions (Figure 2), as long-term use could potentially elicit neuroplastic changes in sensory and motor regions of the brain [144]. This adaptive cortical reorganization could be induced through several VR-related mechanisms explained in this review, such as multisensory integration, virtual embodiment, or manipulation of body representations by virtual avatars.

Our proposal reflects on how immersive virtual reality may influence different stages of the motor behavior decision-making process. The motivational context of VR could enhance the hypoalgesic mechanisms of exercise, both by increasing the activity of the endogenous analgesic system and by changing the valence of the experience towards a positive one. It may serve as a medium to create personalized experiences to promote safe learning in the individual’s relationship with the environment through movement, building self-efficacy in their own body, and disrupting unhelpful cognitive representations, behaviors, and emotional responses to pain [145]. This is key in a patient-centered care approach. 

According to the paradigm outlined in this paper, future research should evaluate the influence on motor behavior and movement-evoked pain (MEP) when experiencing immersive virtual reality to target both responders and non-responders to this intervention.

## Figures and Tables

**Table 1:** Previous theories about chronic pain and its relationship with their bodies and environment.

Model	Contributions
Model of mature organism (MOM)	In 1990, Gifford proposed a complex dynamic view of the patient’s health experience. In his model of the mature organism (MOM), he introduced fundamental pathways in the interactions between the body and the environment through sensory information [10].
The triple network model	The triple network model offers a unified framework to understand the central neurophysiological mechanisms that occur in the experience of pain. First, the influence of the sensory network may modulate the salience network (related to the emotional response to sensory stimuli e.g., suffering). Second, the interrelationship with the default network shapes body self-concept through body embodiment. Thirdly, the response at the level of the executive central network and the sensorimotor network regulates the response through both action and behavior [11].
Embodied Predictive Processing Theory of Pain	This model based on the Enactive Markov Decision Process (EMDP) attempts to explain the embedded complex interaction between perception and action through the sensorimotor system, where motivational factors modulate behavior based on both positive and negative reinforcement learning mechanisms [12]. The model aimed to maintain the functional integrity of the body through the process of prediction error minimization [13].
Enactive-biopsychosocial approach	An enactive-biopsychosocial approach emphasizes that human experiences are influenced by the interactive relationship between the subject and the world, with the brain and body being mediators of that relationship [8]. This framework could help us understand the complexity of the pain experience that is intrinsically embodied and embedded in an environment [14]. This model adds the idea that pain experience incorporates action as a response to the environment, which includes the sensorimotor system. The enactive model shows that pain cannot be reduced to its neurophysiological underpinnings [15].

**Table 2:** Eligibility criteria of literature search.

Inclusion Criteria	Exclusion Criteria
Systematic review and/or meta-analysis assessing the efficacy of virtual reality therapy intervention(s) for chronic pain	Including evaluative, psychological, and/or non-virtual intervention(s)
Systematic review and/or meta-analysis with an adequate description of methodology	Non full text available
Published in a peer reviewed journal	
Published since year 2017	
Written in the English language	

**Table 3:** Properties related to body perception and embodiment.

Concept	Definition
Body image	It refers to implicit cortical maps that encode movement and position of the body
Body embodiment	The experience of our self as the whole set of sensations that emerges from being in, having and controlling a body
Body ownership	It is the feeling that “this body belongs to me”
Body Agency	The perception that “I am the one who caused this action”
Co-location	being in precise place, in time and space.
Body perception disturbance	It refers to an alteration in the size, shape or position of the experienced physical self

## Data Availability

Not applicable.

## Appendix A. Evidence Review of VR in Chronic Pain

**Review**	**Focus of Review**	**Study Details** **Number (N)** **Year** **Target Population**	**Methods** **Database** **Outcomes** **QoE**	**Main Findings**	**Limitations**
Goudmann et al. [19]	Evaluate the effect of VR on several outcome parameters related to the application of VR in patients with chronic pain	2022 N Studies = 41 N participants = 1232 Chronic pain (Fibromyalgia, Low back pain, neck pain, upper limb complex regional pain syndrome and phantom limb pain)	4 Database Risk of bias varied between 8 out of 28 and 25 out of 28 Data from 25 studies were included in the meta-analysis Pain-related Outcomes kinesiophobia and fear, mood, satisfaction, expectations of pain, pain focus, time spent thinking about pain, self-efficacy, emotions, motivation, stress, catastrophizing, acceptability, global impression of change, ownership, and agency Functional outcomes Functional capacity Mobility Neuropsychological functions Experience of VR technology	A total of 23 studies used immersive VR techniques, and 18 used non-immersive techniques. VR intervention in patients with chronic pain had a positive effect on decrease pain, increase mobility and functional capacity. Overall effect of VR on several outcome measurements is not moderated by the type of VR intervention, type of pain or the objective of VR.	None of the included studies qualified for excellent methodological quality. Much heterogeneity is present in studies with VR methodology Authors mainly focused on the primary outcome variables and secondary outcomes were not always described in full detail an asymmetrical plot in experience of VR technology outcomes, which might be interpreted as an indication of publication bias.
Wittkopf et al. [145]	Evaluate the effect of immersive and non-immersive interactive VR on pain perception in patients with a clinical pain condition.	2019 N Studies = 13 N participants = 469 Chronic pain (Low back pain, neck pain, neuropathic pain, phantom limb pain, ankylosing spondylitis, subacromial impingement syndrome and post-mastectomy)	5 databases High risk of bias and small sample sizes. A meta-analysis could not be conducted due to differences in study designs and types of controls. Pain-related Outcomes were included	A total of 5 studies used immersive VR techniques, and 8 used non-immersive techniques. No difference in efficacy in immersive or non-immersive VR intervention. Interactive VRmay reduce pain	High risk of bias and small sample sizes on studies included Much heterogeneity in VR methodology (type, frequency, and duration of VR treatment)
Grassini [146]	Assessment of the efficacy of the use of VR for chronic pain management	2022 N Studies = 9 N participants = 524 Chronic pain (Low back pain and neck pain)	6 databases Low risk of bias and small sample sizes. A meta-analysis could not be conducted due to differences in study designs and types of controls. Pain-related Outcomes Tampa Scale for kinesiophobia (TSK) pain intensity Oswestry dysfunction index (ODI) neck disability index (NDI) were included	VR interventions may be useful for chronic pain management but was not superior to other types of interventions VR could be effective on NDI but no in RMD an TSK	Small number of included studies High heterogeneity was present in most of the outcomes No information about type of VR information A single author has conducted this study
Brea-Gómez et al. [147]	Analyze the effectiveness of VR in chronic low back pain.	2021 N Studies = 14 N participants = 765 Chronic pain (Low back pain)	4 databases Risk of bias varied between 13-27 out 28. Data from 11 studies were included in the meta-analysis Pain-related Outcomes Tampa Scale for kinesiophobia (TSK) Disability Questionnaire (RMDQ) Oswestry dysfunction index (ODI) 10-item Pain Self- Efficacy (10-PSEQ) Pain Catastrophizing Scale (PCS) isokinetic trunk flexion/extension with a dynamometer blood serum levels of stress hormones were included	A total of 2 studies used immersive VR techniques, and 12 used non-immersive techniques. VR can significantly reduce pain intensity and kinesiophobia in patients with chronic low back pain No significant differences were found in disability postintervention.	High heterogeneity between included Differences in the age ranges and in the clinical profile of the participants Small sample sizes on studies included High heterogeneity in VR methodology (type, frequency, and duration of VR treatment)
Gava et al. [136]	Analyze the current evidence regarding the use of games and virtual reality to improve mental health-related outcomes in patients with chronic musculoskeletal pain.	2022 N Studies = 13 N participants = 680 Chronic musculoskeletal pain (back, neck, and shoulder pain; osteoarthritis; fibromyalgia)	6 Database Risk of bias varied between 8 out of 28 and 25 out of 28 Data from 13 studies were included in the meta-analysis Pain-related Outcomes Fear-Avoidance Beliefs Questionnaire (FABQ) Tampa Kinesiophobia Scale (TSK) The Pain Catastrophizing Scale (PCS) Pain Anxiety Symptoms Scale Hospital Anxiety and Depression Scale	A total of 7 studies used immersive VR techniques, and 5 used non-immersive techniques (Exergames) VR intervention in patients with chronic musculoskeletal Pain are superior to other treatments to improve pain-related fear and superior to no treatment to improve anxiety. Gaming was not superior to other treatments or no treatment for improving pain catastrophizing, anxiety, and depression.	Very low or low quality of evidence of studies included Much heterogeneity is present in studies with VR gaming methodology (type, frequency, and duration of VR treatment) Publication bias was not assessed due to the limited number of included Studies.
Ahern et al. [148]	Evaluate effectiveness of VR technology in the management of individuals with acute, subacute, and chronic spinal pain.	2020 N Studies = 7 N participants = 469 Spinal Chronic pain (neck pain, thoracic pain, or low back pain [LBP])	5 databases All 7 of the studies included had a high risk of bias Data from 2 studies were included in the meta-analysis Pain intensity Disability specific function, general health status, future visits to healthcare professionals, return to work, patient satisfaction, adverse events, global perceived effect (GPE), balance, and fear of movement. were included	A total of 1 studies used immersive VR techniques, and 6 used non-immersive techniques. The effect of VR ranged from no statistical significance to clinical significance, depending on the area of the spine being treated, the follow-up period being assessed, and the type of VR used. Difference in effect between VR and other included interventions was often small and not clinically significant higher-quality research on efficacy and effectiveness of VR is needed	The review was limited by the low number of included studies Young population in studies included, results may not be applicable to younger or older populations. Much heterogeneity in VR methodology (type, frequency, and duration of VR treatment)
Mallari et al. [149]	Compare the effectiveness of VR in reducing acute and chronic pain in adults.	2019 N Studies = 20 N participants = No info Chronic pain (Musculoskeletal pain conditions (MSKP), four in neuropathic pain conditions (NP), one in a mixture of musculoskeletal and neuropathic pain conditions (MSKP-NP) and two in an unspecified pain condition (UnP).	3 databases All chronic pain studies had fair to high quality assessment ratings. Data from 3 studies were included in the meta-analysis Pain-related Outcomes Range of motion (ROM), strength, function, balance, and gait	A total of 18 studies used immersive VR techniques, and 2 used non-immersive techniques. VR is an effective tool in reducing chronic pain, specially while the patient is immersed in the VR environment Further research is needed to assess the extent to which one needs to be immersed and present in a virtual environment in order to reduce pain, and the dosage necessary to maintain pain reductions in chronic pain over time.	Significant heterogeneity in study population and pain conditions Much heterogeneity in VR methodology (type, frequency, and duration of VR treatment)
Gumaa et al. [150]	Analyze the effectiveness of VR in chronic low back pain.	2019 N Studies = 19 N participants = 765 Chronic pain (fibromyalgia, rheumatoid arthritis, Musculoskeletal pain conditions)	5 databases Quality Assessment varied between 22–37 out 48. Data from 3 studies were included in the meta-analysis Pain-related Outcomes Functional outcomes Functional capacity Mobility	Evidence of VR effectiveness in individuals with chronic neck pain and shoulder impingement syndrome is promising For fibromyalgia, total knee arthroplasty, and back pain, the evidence of VR effectiveness compared with more traditional exercise is absent or inconclusive	Studies assessed psychosocial outcomes were excluded Heterogeneity in VR and physical therapist interventions as well as outcome measures Small sample sizes on studies included High heterogeneity in VR methodology (type, frequency, and duration of VR treatment)
Virtual Reality (VR); Tampa Scale for kinesiophobia (TSK); Oswestry dysfunction index (ODI); neck disability index (NDI); Roland Morris Disability Questionnaire (RMDQ); 10-item Pain Self-Efficacy (10-PSEQ); Pain Catastrophizing Scale (PCS); low back pain (LBP); Musculoskeletal pain conditions (MSKP); neuropathic pain conditions (NP); Range of motion (ROM).					

## References

[1] Treede R.D., Rief W., Barke A., Aziz Q., Bennett M.I., Benoliel R., Cohen M., Evers S., Finnerup N.B., First M.B. (2015). A classification of chronic pain for ICD-11. Pain.

[2] Treede R.D., Rief W., Barke A., Aziz Q., Bennett M.I., Benoliel R., Cohen M., Evers S., Finnerup N.B., First M.B. (2019). Chronic pain as a symptom or a disease: The IASP Classification of Chronic Pain for the International Classification of Diseases (ICD-11). Pain.

[3] Nicholas M., Vlaeyen J.W.S., Rief W., Barke A., Aziz Q., Benoliel R., Cohen M., Evers S., Giamberardino M.A., Goebel A. (2019). The IASP classification of chronic pain for ICD-11: Chronic primary pain. Pain.

[4] Vlaeyen J.W.S., Haslbeck J.M.B., Sjouwerman R., Peters M.L. (2022). Towards a dynamic account of chronic pain. Pain.

[5] Ilundáin-Agurruza J. (2022). Relational Pain: The Perspective from the Other Side of the Lens. Constr. Found..

[6] Tabor A., Van Ryckeghem D.M.L., Hasenbring M.I. (2020). Pain Unstuck: The Role of Action and Motivation. Clin. J. Pain.

[7] Eccleston C. (2018). Chronic pain as embodied defence: Implications for current and future psychological treatments. Pain.

[8] Stilwell P., Harman K. (2019). An enactive approach to pain: Beyond the biopsychosocial model. Phenomenol. Cogn. Sci..

[9] Miyahara K. (2021). Enactive pain and its sociocultural embeddedness. Phenomenol. Cogn. Sci..

[10] Jones M., Edwards I., Gifford L. (2002). Conceptual models for implementing biopsychosocial theory in clinical practice. Man Ther..

[11] De Ridder D., Vanneste S., Smith M., Adhia D. (2022). Pain and the Triple Network Model. Front. Neurol..

[12] Georgeon O.L., Marshall J.B., Manzotti R. (2013). ECA: An enactivist cognitive architecture based on sensorimotor modeling. Biol. Inspired. Cogn. Archit..

[13] Kiverstein J., Kirchhoff M.D., Thacker M. (2022). An Embodied Predictive Processing Theory of Pain Experience. Rev. Philos. Psychol..

[14] Cormack B., Stilwell P., Coninx S., Gibson J. (2022). The biopsychosocial model is lost in translation: From misrepresentation to an enactive modernization. Physiother. Theory Pract..

[15] Smrdu M. (2022). Kaleidoscope of Pain: What and How Do You See Through It. Constr. Found..

[16] Levin M.F., Weiss P.L., Keshner E.A., Levin M.F., Weiss P.L., Keshner E.A. (2015). Emergence of Virtual Reality as a Tool for Upper Limb Rehabilitation: Incorporation of Motor Control and Motor Learning Principles. Phys. Ther..

[17] Hutting N., Caneiro J.P., Ong’wen O.M., Miciak M., Roberts L. (2022). Patient-centered care in musculoskeletal practice: Key elements to support clinicians to focus on the person. Musculoskelet. Sci. Pract..

[18] Trost Z., France C., Anam M., Shum C. (2021). Virtual reality approaches to pain: Toward a state of the science. Pain.

[19] Goudman L., Jansen J., Billot M., Vets N., De Smedt A., Roulaud M., Rigoard P., Moens M. (2022). Virtual Reality Applications in Chronic Pain Management: Systematic Review and Meta-analysis. JMIR Serious Games.

[20] Slater M. (2018). Immersion and the illusion of presence in virtual reality. Br. J. Psychol..

[21] Harvie D.S., Smith R.T., Martin D., Hirsh A.T., Trost Z. (2022). Editorial: Novel applications of virtual and mixed reality in pain research and treatment. Front. Virtual Real..

[22] Austin P.D. (2022). The Analgesic Effects of Virtual Reality for People with Chronic Pain: A Scoping Review. Pain Med..

[23] Keefe F.J., Huling D.A., Coggins M.J., Keefe D.F., Zachary Rosenthal M., Herr N.R., Hoffman H.G. (2012). Virtual reality for persistent pain: A new direction for behavioral pain management. Pain.

[24] Simón-Vicente L., Rodríguez-Cano S., Delgado-Benito V., Ausín-Villaverde V., Cubo Delgado E. (2022). Cybersickness. A systematic literature review of adverse effects related to virtual reality. Neurología.

[25] Saredakis D., Szpak A., Birckhead B., Keage H.A.D., Rizzo A., Loetscher T. (2020). Factors associated with virtual reality sickness in head-mounted displays: A systematic review and meta-analysis. Front. Hum. Neurosci..

[26] Weech S., Kenny S., Barnett-Cowan M. (2019). Presence and cybersickness in virtual reality are negatively related: A review. Front. Psychol..

[27] Indovina P., Barone D., Gallo L., Chirico A., de Pietro G., Giordano A. (2018). Virtual Reality as a Distraction Intervention to Relieve Pain and Distress During Medical Procedures. Clin. J. Pain.

[28] La Touche R. (2021). Introduciendo la dimensión motora dentro de la conceptualización de la experiencia del dolor. J. MOVE Ther. Sci..

[29] Brady N., McVeigh J.G., McCreesh K., Rio E., Dekkers T., Lewis J.S. (2021). Exploring the effectiveness of immersive Virtual Reality interventions in the management of musculoskeletal pain: A state-of-the-art review. Phys. Ther. Rev..

[30] Kim J., Mawla I., Kong J., Lee J., Gerber J., Ortiz A., Kim H., Chan S.-T., Loggia M.L., Wasan A.D. (2019). Somatotopically-specific primary somatosensory connectivity to salience and default mode networks encodes clinical pain. Pain.

[31] De Ridder D., Adhia D., Vanneste S. (2021). The anatomy of pain and suffering in the brain and its clinical implications. Neurosci. Biobehav. Rev..

[32] Baliki M.N., Mansour A.R., Baria A.T., Apkarian A.V. (2014). Functional reorganization of the default mode network across chronic pain conditions. PLoS ONE.

[33] Gombaut C., Holmes S.A. (2022). Sensorimotor Integration and Pain Perception: Mechanisms Integrating Nociceptive Processing. A Systematic Review and ALE-Meta Analysis. Front. Integr. Neurosci..

[34] Colloca L., Raghuraman N., Wang Y., Akintola T., Brawn-Cinani B., Colloca G.C., Kier C., Varshney A., Murthi S. (2020). Virtual reality: Physiological and behavioral mechanisms to increase individual pain tolerance limits. Pain.

[35] Meulders A. (2019). From fear of movement-related pain and avoidance to chronic pain disability: A state-of-the-art review. Curr. Opin. Behav. Sci..

[36] Den Hollander M., Smeets R.J.E.M., Van Meulenbroek T., Van Laake-Geelen C.C.M., Baadjou V.A., Timmers I. (2022). Exposure in Vivo as a Treatment Approach to Target Pain-Related Fear: Theory and New Insights From Research and Clinical Practice. Phys. Ther..

[37] Boudreau S.A., Farina D., Falla D. (2010). The role of motor learning and neuroplasticity in designing rehabilitation approaches for musculoskeletal pain disorders. Man. Ther..

[38] De Ridder D., Vanneste S., Freeman W. (2014). The Bayesian brain: Phantom percepts resolve sensory uncertainty. Neurosci. Biobehav. Rev..

[39] Orbán G., Wolpert D.M. (2011). Representations of uncertainty in sensorimotor control. Curr. Opin. Neurobiol..

[40] Bach D.R., Dolan R.J. (2012). Knowing how much you don’t know: A neural organization of uncertainty estimates. Nat. Rev. Neurosci..

[41] Sündermann O., Flink I., Linton S.J. (2020). My body is not working right: A cognitive behavioral model of body image and chronic pain. Pain.

[42] Talsma D., Senkowski D., Soto-Faraco S., Woldorff M.G. (2010). The multifaceted interplay between attention and multisensory integration. Trends Cogn. Sci..

[43] Kirsch W., Kunde W. (2022). On the Role of Interoception in Body and Object Perception: A Multisensory-Integration Account. Perspect. Psychol. Sci..

[44] Senkowski D., Höfle M., Engel A.K. (2014). Crossmodal shaping of pain: A multisensory approach to nociception. Trends Cogn. Sci..

[45] Viceconti A., Camerone E.M., Luzzi D., Pentassuglia D., Pardini M., Ristori D., Rossettini G., Gallace A., Longo M.R., Testa M. (2020). Explicit and implicit Own’s body and space perception in painful musculoskeletal disorders and rheumatic diseases: A systematic scoping review. Front. Hum. Neurosci..

[46] Brumagne S., Diers M., Danneels L., Lorimer Moseley G., Hodges P.W. (2019). Neuroplasticity of Sensorimotor Control in Low Back Pain. J. Orthop. Sport. Phys..

[47] Barbosa A.M., José-Jandre dos Reis F., Caseiro M., Barbero M., Falla D., Siriani de Oliveira A. (2021). Clinical evaluation of somatosensory integrity in people with chronic shoulder pain. Musculoskelet. Sci. Pract..

[48] Catley M.J., O’Connell N.E., Berryman C., Ayhan F.F., Moseley G.L. (2014). Is tactile acuity altered in people with chronic pain? A systematic review and meta-analysis. J. Pain.

[49] Vittersø A.D., Halicka M., Buckingham G., Proulx M.J., Bultitude J.H. (2022). The sensorimotor theory of pathological pain revisited. Neurosci. Biobehav. Rev..

[50] Jenkins L.C., Chang W.J., Buscemi V., Liston M., Skippen P., Cashin A.G., McAuley J.H., Schabrun S.M. (2022). Low Somatosensory Cortex Excitability in the Acute Stage of Low Back Pain Causes Chronic Pain. J. Pain.

[51] Don S., Voogt L., Meeus M., De Kooning M., Nijs J. (2017). Sensorimotor Incongruence in People with Musculoskeletal Pain: A Systematic Review. Pain Pract..

[52] Moseley G.L., Flor H. (2012). Targeting cortical representations in the treatment of chronic pain: A review. Neurorehabil. Neural. Repair.

[53] Bushnell M.C., Čeko M., Low L.A. (2013). Cognitive and emotional control of pain and its disruption in chronic pain. Nat. Rev. Neurosci..

[54] Reddan M.C., Wager T.D. (2019). Brain systems at the intersection of chronic pain and self-regulation. Neurosci. Lett..

[55] Martinez-Calderon J., Flores-Cortes M., Morales-Asencio J.M., Luque-Suarez A. (2020). Which Psychological Factors Are Involved in the Onset and/or Persistence of Musculoskeletal Pain? An Umbrella Review of Systematic Reviews and Meta-Analyses of Prospective Cohort Studies. Clin. J. Pain.

[56] Martinez-Calderon J., Matias-Soto J., Luque-Suarez A. (2022). “My Pain Is Unbearable…I Cannot Recognize Myself!” Emotions, Cognitions, and Behaviors of People Living With Musculoskeletal Disorders: An Umbrella Review. J. Orthop. Sport. Phys. Ther..

[57] Ahmadpour N., Randall H., Choksi H., Gao A., Vaughan C., Poronnik P. (2019). Virtual Reality interventions for acute and chronic pain management. Int. J. Biochem. Cell Biol..

[58] Barcatta K., Holl E., Battistutta L., van der Meulen M., Rischer K.M. (2022). When Less Is More: Investigating Factors Influencing the Distraction Effect of Virtual Reality From Pain. Front. Pain Res..

[59] Press C., Taylor-Clarke M., Kennett S., Haggard P. (2004). Visual enhancement of touch in spatial body representation. Exp. Brain Res..

[60] Haggard P., Taylor-Clarke M., Kennett S. (2003). Tactile perception, cortical representation and the bodily self. Curr. Biol..

[61] Longo M.R., Betti V., Aglioti S.M., Haggard P. (2009). Visually induced analgesia: Seeing the body reduces pain. J. Neurosci..

[62] Longo M.R., Iannetti G.D., Mancini F., Driver J., Haggard P. (2012). Linking Pain and the Body: Neural Correlates of Visually Induced Analgesia. J. Neurosci..

[63] Hoffman H.G., Richards T.L., Bills A.R., Van Oostrom T., Magula J., Seibel E.J., Sharar S.R. (2006). Using FMRI to study the neural correlates of virtual reality analgesia. CNS Spectr..

[64] Gold J.I., Belmont K.A., Thomas D.A. (2007). The neurobiology of virtual reality pain attenuation. Cyberpsychol. Behav..

[65] Huynh V., Bekrater-Bodmann R., Fröhner J., Vogt J., Beckerle P. (2019). Robotic hand illusion with tactile feedback: Unravelling the relative contribution of visuotactile and visuomotor input to the representation of body parts in space. PLoS ONE.

[66] Christ O., Reiner M. (2014). Perspectives and possible applications of the rubber hand and virtual hand illusion in non-invasive rehabilitation: Technological improvements and their consequences. Neurosci. Biobehav. Rev..

[67] Kokkinara E., Slater M. (2014). Measuring the Effects through Time of the Influence of Visuomotor and Visuotactile Synchronous Stimulation on a Virtual Body Ownership Illusion. Perception.

[68] Matamala-Gomez M., Diaz Gonzalez A.M., Slater M., Sanchez-Vives M.V. (2019). Decreasing Pain Ratings in Chronic Arm Pain Through Changing a Virtual Body: Different Strategies for Different Pain Types. J. Pain.

[69] Leemhuis E., De Gennaro L., Pazzaglia A.M. (2019). Disconnected Body Representation: Neuroplasticity Following Spinal Cord Injury. J. Clin. Med..

[70] De Haan E.H.F., Dijkerman H.C. (2020). Somatosensation in the Brain: A Theoretical Re-evaluation and a New Model. Trends Cogn. Sci..

[71] Matamala-Gomez M., Donegan T., Bottiroli S., Sandrini G., Sanchez-Vives M.V., Tassorelli C. (2019). Immersive Virtual Reality and Virtual Embodiment for Pain Relief. Front. Hum. Neurosci..

[72] Barra J., Giroux M., Metral M., Cian C., Luyat M., Kavounoudias A., Guerraz M. (2020). Functional properties of extended body representations in the context of kinesthesia. Neurophysiol. Clin..

[73] Von Piekartz H., Paris-Alemany A., Florencio L., De C.F., Peñas L. (2021). Assessment and Brain Training of Patients Experiencing Head and Facial Pain with a Distortion of Orofacial Somatorepresentation: A Narrative Review. Appl. Sci..

[74] Shimada S. (2022). Multisensory and Sensorimotor Integration in the Embodied Self: Relationship between Self-Body Recognition and the Mirror Neuron System. Sensors.

[75] Chancel M., Ehrsson H.H., Ma W.J. (2022). Uncertainty-based inference of a common cause for body ownership. eLife.

[76] Tsakiris M., Longo M.R., Haggard P. (2010). Having a body versus moving your body: Neural signatures of agency and body-ownership. Neuropsychologia.

[77] Pei Y., Zhang Y., Zhu Y., Zhao Y., Zhou F., Huang M., Wu L., Gong H. (2020). Hyperconnectivity and high temporal variability of the primary somatosensory cortex in low-back-related leg pain: An fMRI study of static and dynamic functional connectivity. J. Pain Res..

[78] Haslam B.S., Butler D.S., Moseley G.L., Kim A.S., Carey L.M. (2022). “My Hand Is Different”: Altered Body Perception in Stroke Survivors with Chronic Pain. Brain Sci..

[79] Trojan J., Diers M., Valenzuela-Moguillansky C., Torta D.M.E. (2014). Body, space, and pain. Front. Hum. Neurosci..

[80] Lotze M., Moseley G.L. (2007). Role of distorted body image in pain. Curr. Rheumatol. Rep..

[81] Van Dijk M.T., van Wingen G.A., van Lammeren A., Blom R.M., de Kwaasteniet B.P., Scholte H.S., Denys D. (2013). Neural Basis of Limb Ownership in Individuals with Body Integrity Identity Disorder. PLoS ONE.

[82] Cardini F., Longo M.R. (2016). Congruency of body-related information induces somatosensory reorganization. Neuropsychologia.

[83] Riva G., Wiederhold B.K., Mantovani F. (2019). Neuroscience of Virtual Reality: From Virtual Exposure to Embodied Medicine. Cyberpsychol. Behav. Soc. Netw..

[84] Harvie D.S., Kelly J., Kluver J., Deen M., Spitzer E., Coppieters M.W. (2022). A randomized controlled pilot study examining immediate effects of embodying a virtual reality superhero in people with chronic low back pain. Disabil. Rehabil. Assist. Technol..

[85] Leemhuis E., Giuffrida V., Giannini A.M., Pazzaglia M. (2021). A Therapeutic Matrix: Virtual Reality as a Clinical Tool for Spinal Cord Injury-Induced Neuropathic Pain. Brain Sci..

[86] Senkowski D., Heinz A. (2016). Chronic pain and distorted body image: Implications for multisensory feedback interventions. Neurosci. Biobehav. Rev..

[87] Vartiainen N., Kirveskari E., Kallio-Laine K., Kalso E., Forss N. (2009). Cortical reorganization in primary somatosen-sory cortex in patients with unilateral chronic pain. J. Pain.

[88] Matamala-Gomez M., Maselli A., Malighetti C., Realdon O., Mantovani F., Riva G. (2021). Virtual Body Ownership Illusions for Mental Health: A Narrative Review. J. Clin. Med..

[89] Samad M., Chung A.J., Shams L. (2015). Perception of Body Ownership Is Driven by Bayesian Sensory Inference. PLoS ONE.

[90] Martini M. (2016). Real, rubber or virtual: The vision of “one’s own” body as a means for pain modulation. A narrative review. Conscious Cogn..

[91] Pyasik M., Ciorli T., Pia L. (2022). Full body illusion and cognition: A systematic review of the literature. Neurosci. Biobehav. Rev..

[92] Matamala-gomez M., Nierula B., Donegan T., Slater M., Sanchez-vives M.V. (2020). Manipulating the Perceived Shape and Color of a Virtual Limb Can Modulate Pain Responses. J. Clin. Med..

[93] Kelly J.M., Coppieters M.W., Kluver J., Deen M., Rio E., Harvie D.S. (2022). “It made you feel like you’ve still got it”: Experiences of people with chronic low back pain undertaking a single session of body image training in virtual reality. Physiother. Theory Pract..

[94] Buetler K.A., Penalver-Andres J., Özen Ö., Ferriroli L., Müri R.M., Cazzoli D., Marchal-Crespo L. (2022). “Tricking the Brain” Using Immersive Virtual Reality: Modifying the Self-Perception Over Embodied Avatar Influences Motor Cortical Excitability and Action Initiation. Front. Hum. Neurosci..

[95] Tsakiris M., Prabhu G., Haggard P. (2006). Having a body versus moving your body: How agency structures body-ownership. Conscious Cogn..

[96] Levin M.F., Demers M. (2020). Motor learning in neurological rehabilitation. Disabil. Rehabil..

[97] Breivik H., Collett B., Ventafridda V., Cohen R., Gallacher D. (2006). Survey of chronic pain in Europe: Prevalence, impact on daily life, and treatment. Eur. J. Pain.

[98] Butera K.A., Fox E.J., George S.Z. (2016). Point of View Toward a Transformed Understanding: From Pain and Movement to Pain With Movement. Phys. Ther..

[99] Mccabe C.S., Cohen H., Blake D.R. (2007). Somaesthetic disturbances in fibromyalgia are exaggerated by sensory motor conflict: Implications for chronicity of the disease?. Rheumatology.

[100] Fullwood D., Means S., Merriwether E.N., Chimenti R.L., Ahluwalia S., Booker S.Q. (2021). Toward understanding movement-evoked pain (MEP) and its measurement: A scoping review. Clin. J. Pain.

[101] Othman R., Swain N., Tumilty S., Jayakaran P., Mani R. (2023). Sensitivity to movement-evoked pain, central sensitivity symptoms, and pro-nociceptive profiles in people with chronic shoulder pain: A parallel-group cross-sectional investigation. Pain Pract..

[102] Kantak S.S., Johnson T., Zarzycki R. (2022). Linking Pain and Motor Control: Conceptualization of Movement Deficits in Patients With Painful Conditions. Phys. Ther..

[103] Holmes S.A., Kim A., Borsook D. (2021). The brain and behavioral correlates of motor-related analgesia (MRA). Neurobiol. Dis..

[104] Timmers I., Quaedflieg C.W.E.M., Hsu C., Heathcote L.C., Rovnaghi C.R., Simons L.E. (2019). The interaction between stress and chronic pain through the lens of threat learning. Neurosci. Biobehav. Rev..

[105] Neige C., Mavromatis N., Gagné M., Bouyer L.J., Mercier C. (2018). Effect of movement-related pain on behaviour and corticospinal excitability changes associated with arm movement preparation. J. Physiol..

[106] Karos K., Meulders A., Gatzounis R., Seelen H.A.M., Geers R.P.G., Vlaeyen J.W.S. (2017). Fear of pain changes movement: Motor behaviour following the acquisition of pain-related fear. Eur. J. Pain.

[107] Guccione A.A., Neville B.T., George S.Z. (2019). Optimization of Movement: A Dynamical Systems Approach to Movement Systems as Emergent Phenomena. Phys. Ther..

[108] Van Dieën J.H., Flor H., Hodges P.W. (2017). Low-Back Pain Patients Learn to Adapt Motor Behavior With Adverse Secondary Consequences. Exerc. Sport. Sci. Rev..

[109] Bock O. (2012). Sensorimotor Adaptation. Encyclopedia of the Sciences of Learning.

[110] Rothgangel A., Bekrater-Bodmann R. (2019). Mirror therapy versus augmented/virtual reality applications: Towards a tailored mechanism-based treatment for phantom limb pain. Pain Manag..

[111] La Touche R. (2020). Métodos de representación del movimiento en rehabilitación. Construyendo un marco conceptual para la aplicación en clínica. J. MOVE Ther. Sci..

[112] Cuenca-Martínez F., Reina-Varona Á., Castillo-García J., La Touche R., Angulo-Díaz-Parreño S., Suso-Martí L. (2022). Pain relief by movement representation strategies: An umbrella and mapping review with meta-meta-analysis of motor imagery, action observation and mirror therapy. Eur. J. Pain.

[113] Suso-Martí L., La Touche R., Angulo-Díaz-Parreño S., Cuenca-Martínez F. (2020). Effectiveness of motor imagery and action observation training on musculoskeletal pain intensity: A systematic review and meta-analysis. Eur J. Pain.

[114] Rizzo M., Petrini L., Claudio Del Percio L., Arendt-Nielsen L., Babiloni C. (2022). Neurophysiological Oscillatory Mechanisms Underlying the Effect of Mirror Visual Feedback-Induced Illusion of Hand Movements on Nociception and Cortical Activation. https://europepmc.org/article/ppr/ppr56435.

[115] Cuenca-Martínez F., Suso-Martí L., León-Hernández J.V., La Touche R. (2020). The Role of Movement Representation Techniques in the Motor Learning Process: A Neurophysiological Hypothesis and a Narrative Review. Brain Sci..

[116] Lotze M., Moseley G.L. (2022). Clinical and Neurophysiological Effects of Progressive Movement Imagery Training for Pathological Pain. J. Pain.

[117] Kober S.E., Settgast V., Brunnhofer M., Augsdörfer U., Wood G. (2022). Move your virtual body: Differences and similarities in brain activation patterns during hand movements in real world and virtual reality. Virtual Real..

[118] Ng Y.L., Ma F., Ho F.K., Ip P., Fu K.w. (2019). Effectiveness of virtual and augmented reality-enhanced exercise on physical activity, psychological outcomes, and physical performance: A systematic review and meta-analysis of randomized controlled trials. Comput. Hum. Behav..

[119] Song J.S., Yamada Y., Kataoka R., Wong V., Spitz R.W., Bell Z.W., Loenneke J.P. (2022). Training-induced hypoalgesia and its potential underlying mechanisms. Neurosci. Biobehav. Rev..

[120] Lima L.V., Abner T.S.S., Sluka K.A. (2017). Does exercise increase or decrease pain? Central mechanisms underlying these two phenomena. J. Physiol..

[121] Wewege M.A., Jones M.D. (2021). Exercise-Induced Hypoalgesia in Healthy Individuals and People With Chronic Musculoskeletal Pain: A Systematic Review and Meta-Analysis. J. Pain.

[122] Carey C., Naugle K.E., Aqeel D., Ohlman T., Naugle K.M. (2017). Active Gaming as a Form of Exercise to Induce Hypoalgesia. Games Health J..

[123] Evans E., Naugle K.E., Ovispo A., Kaleth A.S., Arnold B., Naugle K.M. (2021). Active Virtual Reality Games Reduce Pain Sensitivity in Young, Healthy Adults. Front. Virtual Real..

[124] Wender C.L.A., Ahn S.J., O’Connor P.J. (2019). Interactive Virtual Reality Reduces Quadriceps Pain during High-Intensity Cycling. Med. Sci. Sport. Exerc..

[125] Kami K., Tajima F., Senba E. (2022). Brain Mechanisms of Exercise-Induced Hypoalgesia: To Find a Way Out from “Fear-Avoidance Belief”. Int. J. Mol. Sci..

[126] Nijs J., Lluch Girbés E., Lundberg M., Malfliet A., Sterling M. (2015). Exercise therapy for chronic musculoskeletal pain: Innovation by altering pain memories. Man. Ther..

[127] Vlaeyen J.W.S., Crombez G. (2020). Behavioral Conceptualization and Treatment of Chronic Pain. Annu. Rev. Clin. Psychol..

[128] Seymour B. (2019). Pain: A Precision Signal for Reinforcement Learning and Control. Neuron.

[129] Schlitt F., Schmidt K., Merz C.J., Wolf O.T., Kleine-borgmann J. (2022). Impaired pain-related threat and safety learning in patients with chronic back pain. Pain.

[130] Lin C., Zhuo S., Peng W. (2022). Ongoing pain facilitates emotional decision-making behaviors. Brain Sci. Adv..

[131] Borsook D., Edwards R., Elman I., Becerra L., Levine J. (2013). Pain and analgesia: The value of salience circuits. Prog. Neurobiol..

[132] De Baets L., Matheve T., Timmermans A. (2020). The Association between Fear of Movement, Pain Catastrophizing, Pain Anxiety, and Protective Motor Behavior in Persons with Peripheral Joint Conditions of a Musculoskeletal Origin: A Systematic Review. Am. J. Phys. Med. Rehabil..

[133] Christe G., Benaim C., Luthi F., Jolles B.M., Favre J. (2022). Reduction in pain-related fear is not associated with improvement in spinal biomechanics but with decrease in movement-evoked pain in patients with chronic low back pain. Pain Pract..

[134] Matheve T., De Baets L., Bogaerts K., Timmermans A. (2019). Lumbar range of motion in chronic low back pain is predicted by task-specific, but not by general measures of pain-related fear. Eur. J. Pain.

[135] Vachon-Presseau E., Centeno M.V., Ren W., Berger S.E., Tétreault P., Ghantous M., Baria A., Farmer M., Baliki M.N., Schnitzer T.J. (2016). The Emotional Brain as a Predictor and Amplifier of Chronic Pain. J. Dent. Res..

[136] Gava V., Fialho H.R.F., Calixtre L.B., Barbosa G.M., Kamonseki D.H. (2022). Effects of Gaming on Pain-Related Fear, Pain Catastrophizing, Anxiety, and Depression in Patients with Chronic Musculoskeletal Pain: A Systematic Review and Meta-Analysis. Games Health J..

[137] Wang S., Sun J., Yin X., Li H. (2022). Effect of virtual reality technology as intervention for people with kinesiophobia: A meta-analysis of randomised controlled trials. J. Clin. Nurs..

[138] Chen Q.L., Heinricher M.M. (2019). Descending Control Mechanisms and Chronic Pain. Curr. Rheumatol. Rep..

[139] Coninx S., Stilwell P. (2021). Pain and the field of affordances: An enactive approach to acute and chronic pain. Synthese.

[140] Yarossi M., Mangalam M., Naufel S., Tunik E. (2021). Virtual Reality as a Context for Adaptation. Front. Virtual Real..

[141] Caneiro J.P., Smith A., Bunzli S., Linton S., Moseley G.L., O’Sullivan P. (2021). From Fear to Safety: A Roadmap to Recovery from Musculoskeletal Pain. Phys. Ther..

[142] Osiurak F., Rossetti Y., Badets A. (2017). What is an affordance? 40 years later. Neurosci. Biobehav. Rev..

[143] Coninx S., Ray B.M., Stilwell P. (2023). Unpacking an affordance-based model of chronic pain: A video game analogy. Phenomenol. Cogn. Sci..

[144] Cheung K.L., Tunik E., Adamovich S.V., Boyd L.A. (2014). Neuroplasticity and Virtual Reality. Virtual Reality for Physical and Motor Rehabilitation.

[145] Wittkopf P.G., Lloyd D.M., Coe O., Yacoobali S., Billington J. (2020). The effect of interactive virtual reality on pain perception: A systematic review of clinical studies. Disabil. Rehabil..

[146] Grassini S. (2022). Virtual Reality Assisted Non-Pharmacological Treatments in Chronic Pain Management: A Systematic Review and Quantitative Meta-Analysis. Int. J. Environ. Res. Public Health.

[147] Brea-Gómez B., Torres-Sánchez I., Ortiz-Rubio A., Calvache-Mateo A., Cabrera-Martos I., López-López L., Valenza M.C. (2021). Virtual Reality in the Treatment of Adults with Chronic Low Back Pain: A Systematic Review and Meta-Analysis of Randomized Clinical Trials. Int. J. Environ. Res. Public Health.

[148] Ahern M.M., Dean L.V., Stoddard C.C., Agrawal A., Kim K., Cook C.E., Narciso Garcia A. (2020). The Effectiveness of Virtual Reality in Patients With Spinal Pain: A Systematic Review and Meta-Analysis. Pain Pract..

[149] Mallari B., Spaeth E.K., Goh H., Boyd B.S. (2019). Virtual reality as an analgesic for acute and chronic pain in adults: A systematic review and meta-analysis. J. Pain Res..

[150] Gumaa M., Youssef A.R. (2019). Is Virtual Reality Effective in Orthopedic Rehabilitation? A Systematic Review and Meta-Analysis. Phys. Ther..

